# Preparation of CuCrO_2_ Hollow Nanotubes from an Electrospun Al_2_O_3_ Template

**DOI:** 10.3390/nano9091252

**Published:** 2019-09-03

**Authors:** Hsin-Jung Wu, Yu-Jui Fan, Sheng-Siang Wang, Subramanian Sakthinathan, Te-Wei Chiu, Shao-Sian Li, Joon-Hyeong Park

**Affiliations:** 1 Department of Materials and Mineral Resources Engineering, National Taipei University of Technology, 1, Sec. 3, Zhongxiao E. Rd., Taipei 10608, Taiwan; 2 School of Biomedical Engineering, Taipei Medical University, No. 250, Wuxsing Street, Taipei 11031, Taiwan; 3 Graduate Institute of Biomedical Optomechatronics, College of Biomedical Engineering, Taipei Medical University, No. 250, Wuxing Street, Taipei 11031, Taiwan; 4 Birck Nanotechnology Center, Purdue University, West Lafayette, IN 47907, USA

**Keywords:** electrospinning, CuCrO_2_, hollow nanotube, Al_2_O_3_ template, one-dimensional structures

## Abstract

A hollow nanostructure is attractive and important in different fields of applications, for instance, solar cells, sensors, supercapacitors, electronics, and biomedical, due to their unique structure, large available interior space, low bulk density, and stable physicochemical properties. Hence, the need to prepare hollow nanotubes is more important. In this present study, we have prepared CuCrO_2_ hollow nanotubes by simple approach. The CuCrO_2_ hollow nanotubes were prepared by applying electrospun Al_2_O_3_ fibers as a template for the first time. Copper chromium ions were dip-coated on the surface of electrospun-derived Al_2_O_3_ fibers and annealed at 600 °C in vacuum to form Al_2_O_3_-CuCrO_2_ core-shell nanofibers. The CuCrO_2_ hollow nanotubes were obtained by removing Al_2_O_3_ cores by sulfuric acid wet etching while preserving the rest of original structures. The structures of the CuCrO_2_-coated Al_2_O_3_ core-shell nanofibers and CuCrO_2_ hollow nanotubes were identified side-by-side by X-ray diffraction, field emission scanning electron microscopy, and transmission electron microscopy. The CuCrO_2_ hollow nanotubes may find applications in electrochemistry, catalysis, and biomedical application. This hollow nanotube preparation method could be extended to the preparation of other hollow nanotubes, fibers, and spheres.

## 1. Introduction

One-dimensional (1D) nanostructure materials such as nanotubes, nanobelts, and nanofibers have attracted wide interest in nanoscience and technology [1]. Regulating the size and shape of synthesized nanomaterials is of great technological interest nowadays. Particularly, hollow nanostructures have received considerable attention due to their high surface areas and structural uniqueness, thus they have been extensively applied in many fields, such as sensors, dye-sensitized solar cells, catalysts, supercapacitors, photoelectrochemical cells, electronics, and biomolecule devices. Hence, different approaches have been used in the development of hollow nanotubes and nanofibers for large-scale synthesis [2,3]. One of such structural approaches is electrospinning which has been widely applied to synthesize nanofibers from a variety of oxide materials [4]. 

Electrospinning is a fiber formation method that uses self-repulsion effect, which induces an electrostatic charge on a precursor material to stretch the liquid in an electric field into fiber structure. The dimension of fiber diameter ranges from tens nanometer to few micrometers [5]. In the past few years, it has been an effective method to prepare polymer-based nano- or microfibers. Different kinds of polymers have been successfully electrospun from melts or solutions into ultrathin fibers [6]. Up to date, the preparation of nanofibers with solid cross-sections has been studied [7,8]. 

P-type transparent conducting oxides with delafossite structure has been demonstrated with potential applications in various fields including organic photovoltaic (OPV) devices [9], perovskite solar cells [10], antibacterial surface [11], gas sensors [12], solid propellants [13], etc. The delafossite structure of copper-based catalysts also has great importance in catalytic steam reforming of methanol to hydrogen production and heterogeneous catalysis for chlorine production due to their high thermal stability, fine porous structure, high surface area, high selectivity, and excellent activity at low temperature. Besides, copper delafossite materials are more stable than Ru, Pd, Au, and Pt catalyst at the steam reforming process [14,15,16]. Cu-based delafossites have been reported including CuAlO_2_ [17], CuFeO_2_ [18], CuGaO_2_ [19], CuInO_2_ [20], CuScO_2_ [21], CuCrO_2_ [22], and Mg-doped CuCrO_2_ [23,24]. The chemical formula of delafossite structure is that of a ternary oxide A^+^B^+3^O_2_. According to the report, the delafossite structure of CuCrO_2_ has a wide bandgap of 3.1 eV and highest conductivity among all types of semiconductors [25]. Hence, CuCrO_2_ and CuAlO_2_ have drawn considerable attention in optoelectronic devices [26,27]. The delafossite material consists of two alternating sheets: a planar layer of triangular-patterned cations (A) and a layer of edge-sharing BO_6_ octahedrons flattened with respect to the c-axis. Depending on the orientation of layer stacking, two polytypes of delafossite oxide can be created. Considering the morphological effects, catalyst with hollow tube structure shows very promising potential because of the highly selective catalytic reaction. For example, ZSM-5/SiO_2_ hollow structure catalyst selectively increases the paraxylene from the 24% to 89.6% in xylene in methanol-to-aromatics conversion [28]. A single-wall carbon nanotube/iron tetraphenyl porphyrin composite sensor shows a selectively high response toward xylene among benzene and toluene [29]. Carbon nanotube pores (CNTP) show potential to be used as next-generation water purification technologies because CNTP provides high selectivity of water and anions [30]. Further, a porous hollow tube CeO_2_/Au@SiO_2_ nanocatalyst exhibited excellent catalytic activity toward 4-nitrophenol reduction [31]. Platinum (Pt) functionalized NiO hollow tube exhibited remarkable selectivity of C_2_H_5_OH sensing against CO and H_2_ gases [32]. The hollow structure of CuO@SiO_2_ exhibits excellent catalytic activities toward CO and NO oxidation compared with individual CuO and SiO_2_ [33]. Besides, carbon nanotube catalyst could raise the selectivity of H_2_ production rather than CO [34].

However, nanotube with hollow cross-sections are challenging to fabricate because of multi-step treatments (e.g., a template process) or specially designed instrumentation facilities (e.g., for co-electrospinning with coaxial capillaries) [35]. Nanofiber (7.85 m^2^/g) [36] or nanopowder structures (30.92 m^2^/g) [37], such as hollow nanotubes (136 m^2^/g), have a higher surface-to-volume ratio and higher porosity, which are favorable for adsorption in catalysis [38]. Hence, developing a simple approach to obtain hollow nanotubes is of great importance. [36,39]. In this study, the main objective was to explore the use of Al_2_O_3_ microfibers as a template to prepare a core-shell structure of Al_2_O_3_-CuCrO_2_ by immersion in Cu-Cr-O precursor solution. The alumina structure was then removed by etching in H_2_SO_4_ to form the CuCrO_2_ hollow nanotubes.

## 2. Materials and Methods 

**Figure 1:**
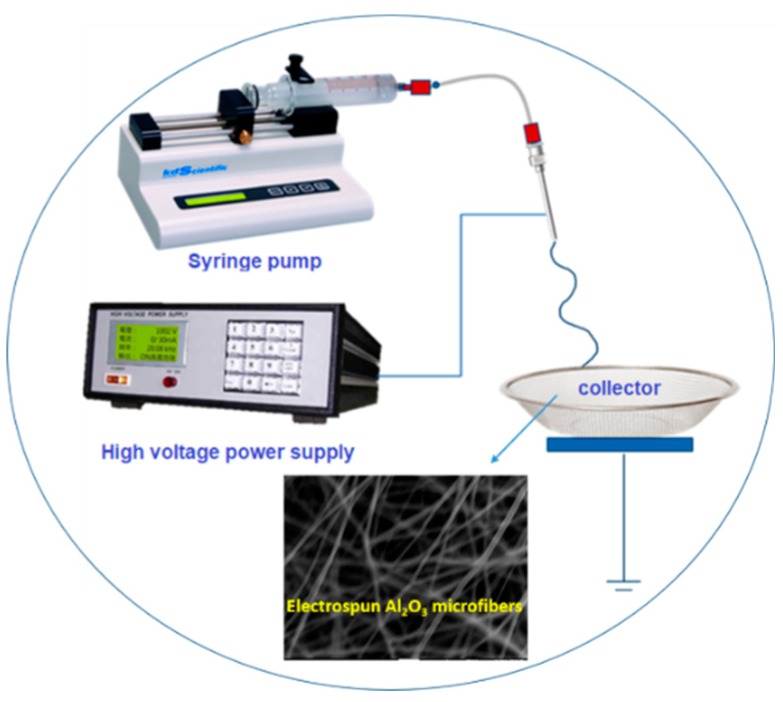
Schematic illustration of electrospinning preparation of as-spun fiber.

All the high-purity chemicals used in this experiment were obtained from Sigma Chemical Co, Taiwan. The electrospun Al_2_O_3_ microfibers precursor was prepared by the electrospinning method. Typically, the precursor solution was prepared by dissolving aluminum nitrate (Al(NO_3_)_3_ 9H_2_O) into 14.4 mL of dimethylformamide (DMF) solvent to make a 0.04 M metal source solution. Then, 2.4 g polyvinylpyrrolidone (Mw = 1,300,000) was mixed into the aforementioned prepared metal source solution followed by constant stirring for 6 h. Finally, a viscous gel-like precursor solution of Al_2_O_3_ was obtained. The Al_2_O_3_ precursor solution was loaded into a horizontal programmable syringe pump. A schematic image of the fundamental electrospinning process is illustrated in Figure 1. An ordinary electrospinning set-up, a high-voltage source is combined with the metallic needle, which is connected to a syringe pump. This syringe pump was connected with Teflon tube (length = 125 mm, diameter = 4.2 mm) for conventional electrospinning setup. During the electrospinning process, the precursor solution was placed in a 10 mL syringe equipped with a stainless steel needle (ID = 0.5 mm). A voltage of 20 kV was applied to the stainless steel needle tip, and the collector was fixed at a distance of 16 cm from the needle tip with the flow controlled at 0.02 mL/h. The electrospun Al_2_O_3_ precursor was distributed uniformly over the collector to form Al_2_O_3_ precursor fibers (Step 1). After the electrospinning, the electrospun Al_2_O_3_ precursor fibers were heated at a rate of 5 °C/min to the annealing temperature of 600 °C in a high-temperature furnace at air atmosphere and then held at that temperature for 2 h, after which Al_2_O_3_ nanofibers were formed (Step 2) and the diameter of the Al_2_O_3_ nanofibers is <100 nm.

### 2.1. Preparation of CuCrO_2_ Hollow Nanotube

**Figure 2:**
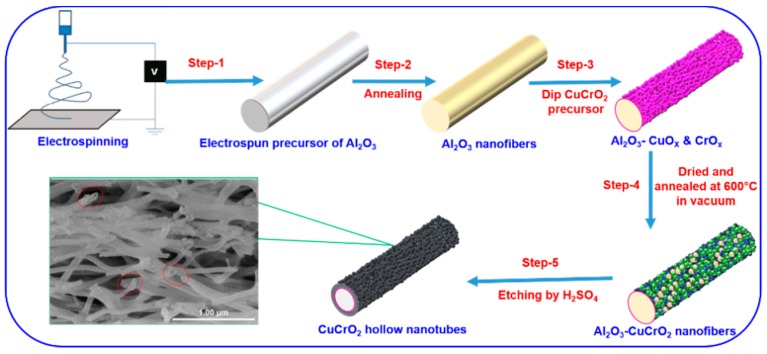
Schematic illustration of CuCrO_2_ hollow nanotubes fabrication process.

Copper (II) acetate, chromium (III) acetate, and ethanolamine were dissolved in ethylene glycol monomethyl ether (30 mL) to obtain 0.2 M precursor. The prepared solution was stirred for 24 h to obtain a well-mixed solution without impurities. Al_2_O_3_ microfibers were dipped in Cu-Cr-O ion solution up to 3 sec to deposit Cu-Cr-O ions on the fiber surfaces and form an Al_2_O_3_-Cu-Cr-O core (Step 3). The Cu-Cr-O ions deposited on Al_2_O_3_ fibers were dried at 80 °C on a hotplate for 2 min. Then the coated fibers were annealed at 600 °C in vacuum (Step 4). After that, the prepared nanofibers were etched with 2 M H_2_SO_4_ to remove the Al_2_O_3_ and other minor impurities from the fibers (Step 5) [39]. The nanofibers were repeatedly rinsed with DI water and a centrifuge was used to separate the liquid and fibers. Finally, the collected nanofibers were dried in an oven at 80 °C to form CuCrO_2_ hollow nanotube (Figure 2).

### 2.2. Characterization

The crystallized phase of Al_2_O_3_ microfibers and CuCrO_2_ hollow nanotubes was characterized with an X-ray diffractometer (XRD, D_2_ Phaser, Bruker) with Cu Kα radiation (λ = 0.15418 nm) from 20° to 80°, a working voltage of 30 kV, and current of 10 mA. The thermal decomposition behavior of the as-spun fibers was identified using a thermogravimetric analysis/differential scanning calorimeter (TGA/DSC, STA 449 F5, NETZSCH) at a heating rate of 10 °C/min. The surface morphology and structure of the nanofibers were observed by field emission scanning electron microscopy (FE-SEM, Hitachi S-4700) SEM 15 kV, 10 cm SEI detector, and nanotubes were identified by transmission electron microscopy (TEM, JEM-2100F, JEOL) operated at a working voltage of 200 kV, working current was10 μA and chamber was about 1.0 × 10^−6^ to 3.0 × 10^−6^ torr. The composition hollow nanotubes were confirmed by JOEL JEM2100F type scanning transmission electron microscope (STEM) attached with an energy dispersive spectrometer (EDS).

## 3. Results

### 3.1. TGA Analysis

**Figure 3:**
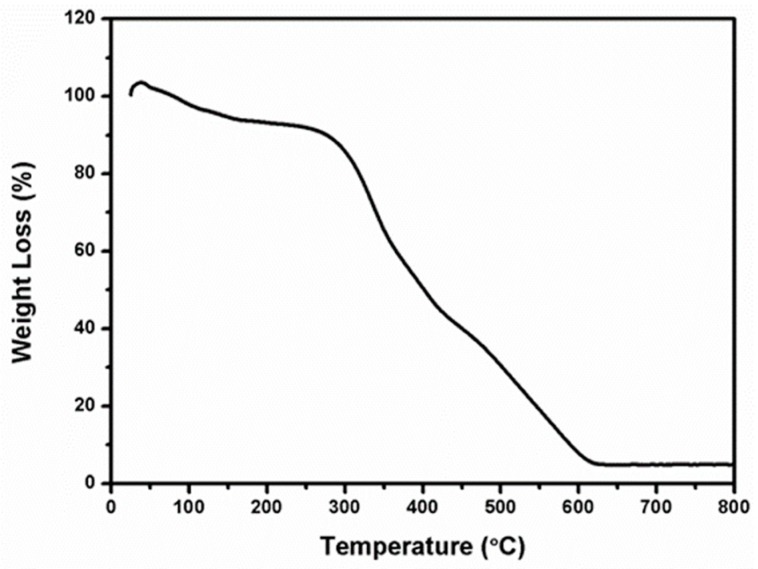
Thermogravimetric-derivative thermal analysis of as-spun Al_2_O_3_ precursor microfibers recorded in air at a heating rate of 10 °C/min.

The TGA/DSC analysis of the Al_2_O_3_ electrospun fibers studied at a heating rate of 10 °C/min in air is shown in Figure 3. Two discrete regions of electrospun fibers weight loss occurred at about 135 °C and 300 °C. The weight loss at around 135 °C could be attributed to DMF solvent. Exothermic peaks at 300 °C with a large weight loss of ~80% corresponded to the decomposition of nitrate, PVP polymer, and other minor organic constituents during the burning combustion. For temperature higher than 600 °C, there was almost no change in the TGA curve, which confirmed that the complete decomposition of organic materials and polymer during the formation of Al_2_O_3_ fibers [40,41,42,43]. 

### 3.2. X-ray Diffraction Investigation

**Figure 4:**
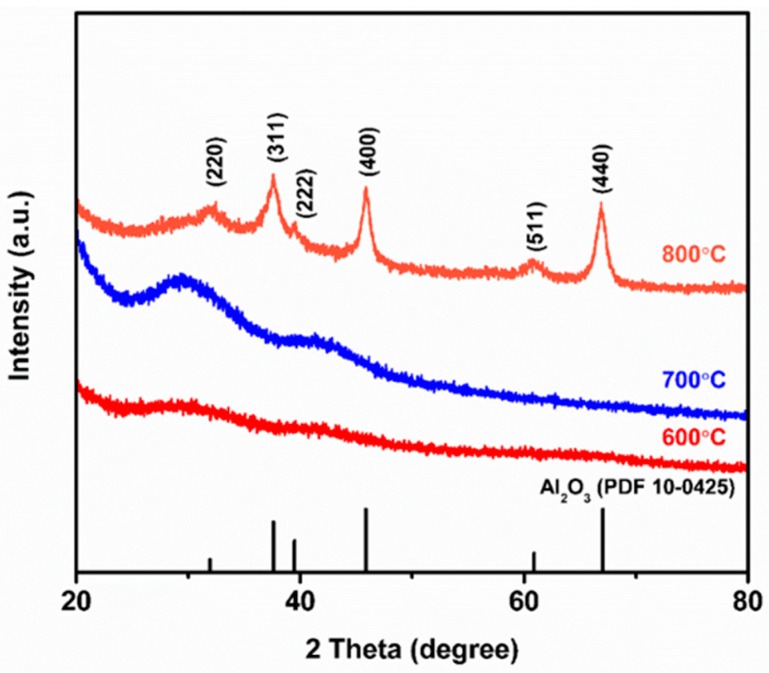
XRD patterns of electrospun Al_2_O_3_ precursor fibers annealed for 2 h in the air at various temperatures (600 °C–800 °C).

Figure 4 shows the XRD analysis of annealed Al_2_O_3_ fibers prepared by electrospinning method. The Al_2_O_3_ fibers were fabricated following the process mentioned in the last section with thermal annealing at elevated temperature for 2 h. We found no distinct diffraction peak for the as-spun fibers, but after the fibers were annealed at 600 °C, a clear amorphous phase was found. The XRD pattern indicated that the Al_2_O_3_ fibers became crystallized when the annealing temperature was over 800 °C [44].

**Figure 5:**
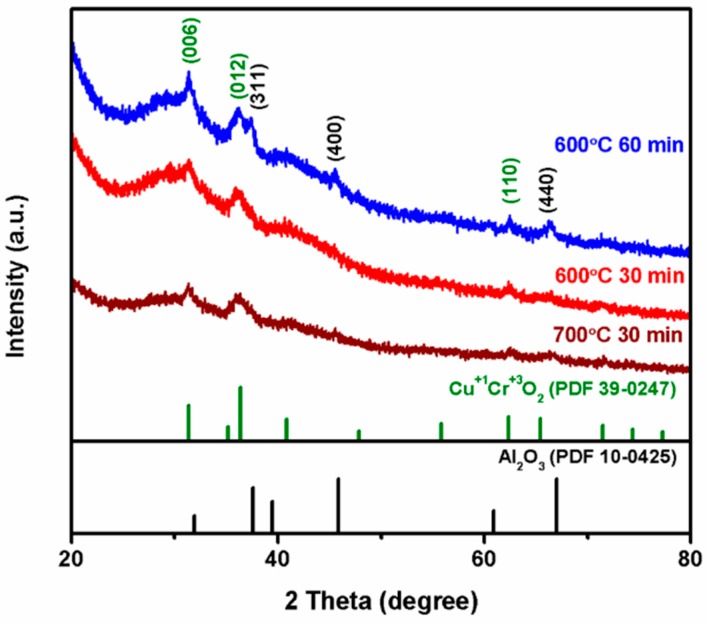
XRD patterns of Al_2_O_3_ microfibers with copper chromium oxide deposited on the surfaces after annealing at 600 °C and 700 °C in vacuum.

Figure 5 shows the XRD pattern of Al_2_O_3_ fibers with copper chromium ions deposited on the surfaces after annealing in vacuum at 600 °C for 30 min and 60 min, and at 700 °C for 30 min. The fibers were composed of an Al_2_O_3_ core and the copper chromium ion solution. The XRD studies show the peaks of Al_2_O_3_ for the fibers annealed at 600 °C for 60 min. It is presumed that the prolonged annealing time caused the crystallization of alumina [39,44].

**Figure 6:**
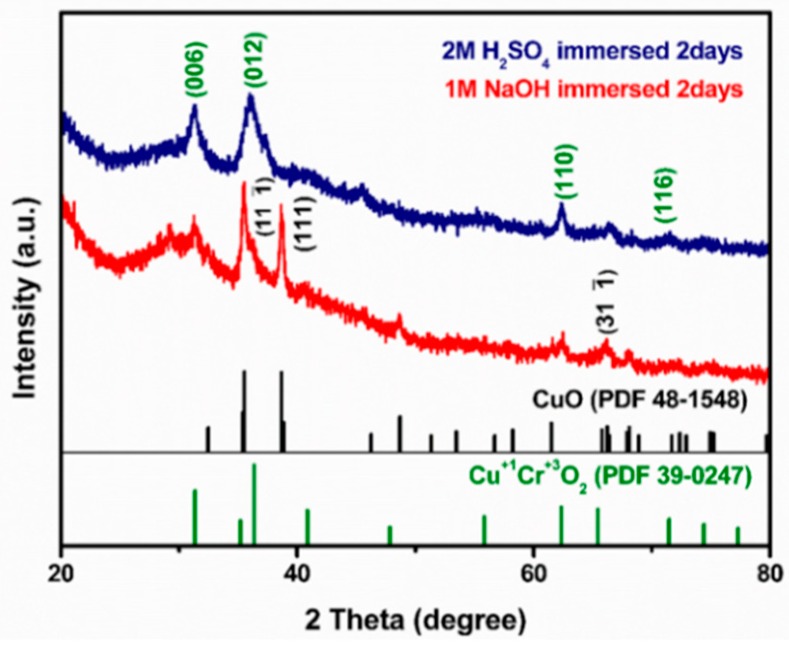
XRD patterns of Al_2_O_3_ microfibers with copper chromium oxide deposited on the surfaces after annealing at 600 °C in vacuum followed by leaching with 2M H_2_SO_4_ and NaOH solution.

Figure 6 shows the XRD pattern of Al_2_O_3_ fibers with copper chromium ion solution deposited on the surfaces after annealing at 600 °C for 30 min in vacuum followed by leaching with 2M H_2_SO_4_ solution due to the strong acid and without the formation of impurities. That solution was employed because Al_2_O_3_ is an amphoteric oxide and reacts with both acid and alkaline solutions. From comparing Figure 6 with Figure 5, it is clear that the main phase of CuCrO_2_ can be clearly seen in the XRD pattern after the acid immersion. For comparison, NaOH solution was also used to remove alumina cores. As can be seen from the figures, after immersion of the fibers in NaOH solution, only the CuO phase remain while the chromium oxide disappeared. Therefore, we concluded that Al_2_O_3_ fibers with copper chromium ion solution deposited on the surfaces could be treated with 2M H_2_SO_4_ solution and DI water to obtain CuCrO_2_ hollow nanotube [39].

### 3.3. SEM Analysis

**Figure 7:**
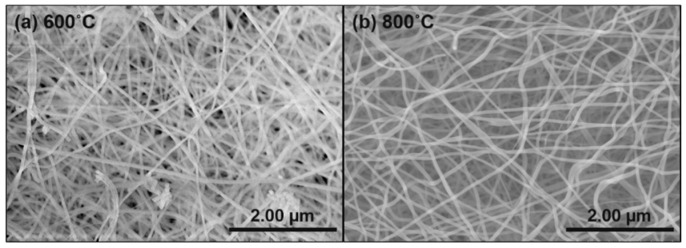
SEM images of electrospun Al_2_O_3_ microfibers annealed for 2 h at (**a**) 600 °C and (**b**) 800 °C.

The SEM micrographs of as-spun Al_2_O_3_ precursor fibers have fine cylindrical with smooth surface morphology and shows in Scheme 1 [41]. Besides, the SEM image of Al_2_O_3_ electrospun fibers annealed for 2 h in air at 600 °C and 800 °C are presented in Figure 7. The morphology of the fibers reveals that the Al_2_O_3_ fibers have continuous, one-dimensional structure and that the diameter of each Al_2_O_3_ fiber is <100 nm. The morphology and dimension of Al_2_O_3_ fibers are essentially similar in the case of annealing temperature of 600 °C and the counterpart in 800 °C. 

**Figure 8:**
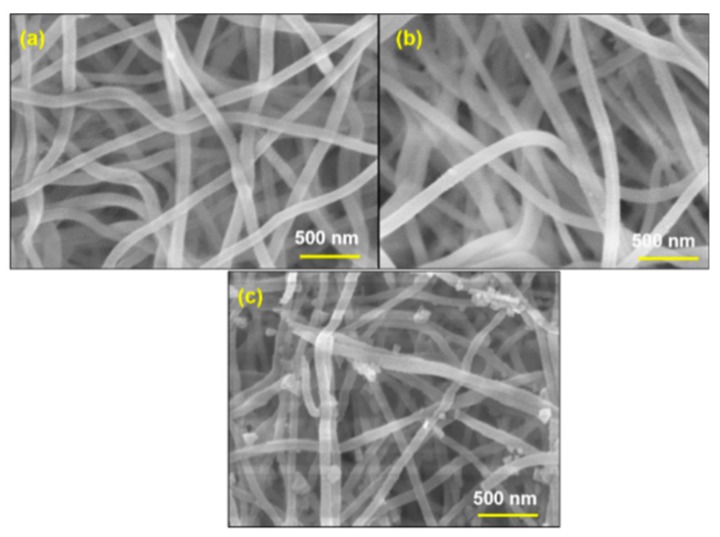
SEM images of Al_2_O_3_-CuCrO_2_ nanofibers annealed at 600 °C for (**a**) 30 min, (**b**) 60 min, and at 700 °C for (**c**) 30 min.

Figure 8 shows the morphology of Al_2_O_3_ fibers immersed in copper chromium ion solution and then dried for 2 min at 80 °C on a hotplate. After that, the Al_2_O_3_-CuCrO_2_ fibers were annealed in vacuum at 600 °C for 30 min (Figure 8a) and 60 min (Figure 8b), and at 700 °C for 30 min (Figure 8c). The surfaces of the fibers are smooth, and there is no specific change compared with calcined amorphous Al_2_O_3_ fibers. The copper chromium ion precursor solution, composed of mixed copper acetate, chromium acetate, and ethanolamine, was dissolved in ethylene glycol monomethyl ether.

**Figure 9:**
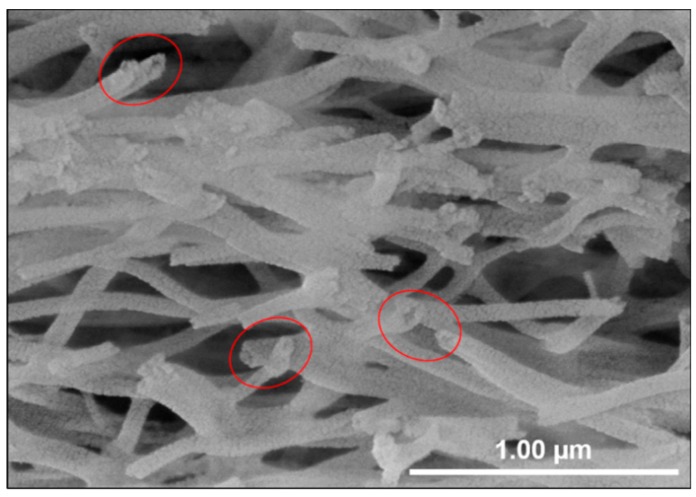
SEM images of CuCrO_2_ hollow nanotubes after removal of Al_2_O_3_ core and impurities by 2M H_2_SO_4_ for 2 days and DI water.

Figure 9 shows a SEM image of Al_2_O_3_-CuCrO_2_ nanofibers after immersion in 2M H_2_SO_4_ and oven-drying at 80 °C for 1 day. As can be seen from the SEM morphology, there is a hollow-like structure at the tip of the CuCrO_2_ nanotubes etched by 2M H_2_SO_4_. It was inferred that the Al_2_O_3_ core was mostly removed by the H_2_SO_4_ solution and remaining impurities were removed by DI water. 

### 3.4. TEM Analysis

**Figure 10:**
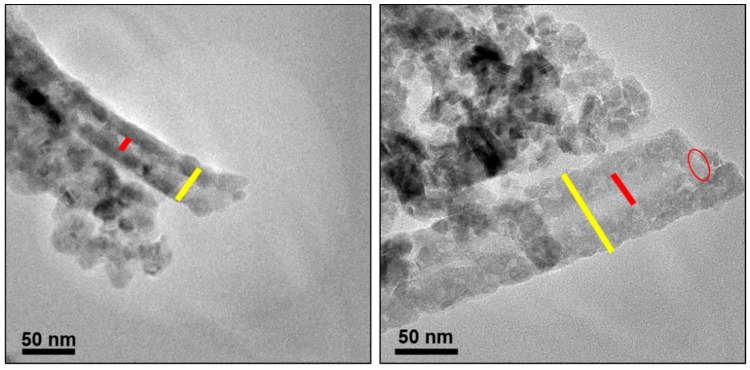
TEM images of the CuCrO_2_ hollow nanotubes.

To identify the structure of the CuCrO_2_ hollow nanotubes synthesized by annealing and followed by chemical etching, TEM was used to further confirm the hollow structures of the nanotubes. The nanotubes were formed by using Al_2_O_3_ fiber as a template and depositing copper chromium ions on the tube surfaces so that the inner core was Al_2_O_3_. As shown in TEM image in Figure 10, the inner template of Al_2_O_3_ was completely etched away by 2M H_2_SO_4_ solution. The inner diameter of the nanotubes was about 70 nm, which is consistent with the diameter of Al_2_O_3_ fiber. The tube wall which consists of CuCrO_2_ features a thickness of several tens of nanometer [39]. These results indicate that the chemical etching method was successful in making CuCrO_2_ hollow nanotubes. Based on previous report, CuCrO_2_ hollow nanotubes have more porous cavity than none-hollow CuCrO_2_ nanofibers due to annealing condition [10].

### 3.5. STEM Analysis

**Figure 11:**
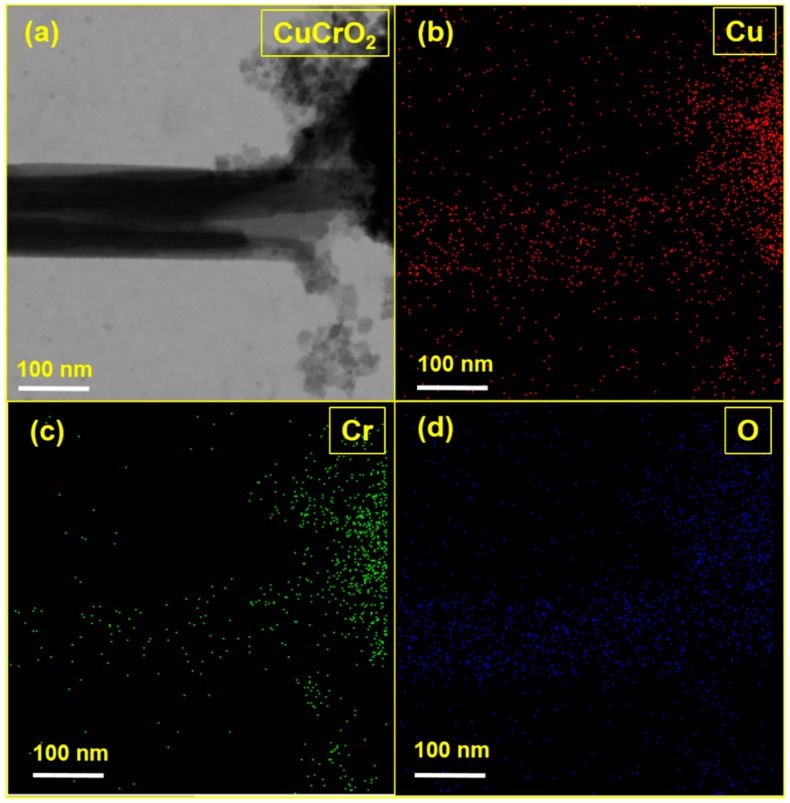
(**a**) STEM image of the CuCrO_2_ hollow nanotube, (**b**) Cu, (**c**) Cr, (**d**) O.

Figure 11a shows a STEM image of CuCrO_2_ hollow nanotube formed by annealing and chemical etching. The average diameter of the CuCrO_2_ hollow nanotube was about 100 nm and that of the center hollow was approximately 20 nm. These results exhibit that the chemical etching method succeeded in producing hollow nanotube. The STEM-EDS signals of CuCrO_2_ nanotube showed the presence of (Figure 11b) Cu, (Figure 11c) Cr, and (Figure 11d) O. Besides, the STEM-EDS spectrum showed higher numbers of atoms present in the tube edge than inside the cavity, which clearly shows the successful formation of the CuCrO_2_ hollow nanotubes.

## 4. Conclusions

CuCrO_2_ hollow nanotubes were successfully prepared by our proposed method using electrospun Al_2_O_3_ fiber as core template. The amorphous Al_2_O_3_ fibers were prepared by annealing the as-spun alumina precursor fibers at 600 °C for 2 h. These continuous and one-dimensional fibers were then deposited with CuCrO_2_ precursor and formed CuCrO_2_ cladding layer by thermal annealing at 600 °C for 30 min. After removing amorphous Al_2_O_3_ core fibers by using H_2_SO_4_, CuCrO_2_ nanotubes with an inner diameter of 70 nm and tube wall thickness of 30 nm were obtained. This work demonstrated a simple solution-based approach for the synthesis of oxide nanotubes and could be further extended to synthesize oxide materials with various complicated hollow structures.
